# How to Sterilize Cotton

**Published:** 1896-06

**Authors:** 


### How to Sterilize Cotton.

A rather ingenious plan for sterilizing cotton is referred to in a French contemporary. A piece of cotton is taken, twisted on a stick or a piece of wood, and dipped into a saturated alcoholic solution of boracic acid for a moment or so. It is then withdrawn from the solution, and a light is applied to it, as the result of which the alcohol burns out, while the boracic acid prevents the cotton from burning. Five seconds are enough; as soon as the flame turns green it is extinguished. The cotton remains white, dry, warm, but absolutely sterilized.—*Med. Press and Circular.*

